# Post-Dural Puncture Headache

**DOI:** 10.5812/aapm.3610

**Published:** 2012-04-01

**Authors:** Marc Wrobel, Thomas Volk

**Affiliations:** 1 Department of Anaesthesiology, Intensive Care Medicine and Pain Therapy, University of Saarland, Homburg, Germany

**Keywords:** Post-Dural Puncture Headache, Anesthesia, Spinal, Spinal Puncture

Dear Editor,

August Bier’s first report of spinal anesthesia in August 1898 impressively described the development of a postdural puncture headache (PDPH) (1). When asked for complications of spinal anesthesia today, patients often respond with PDPH. PDPH is defined as a constant headache that worsens in the sitting or upright position following lumbar puncture (LP). Its incidence after spinal anesthesia in obstetric anesthesia is 1% to 6% (2) and 30% to 50% after a diagnostic LP (3) and can reach 80% after inadvertent LP during epidural obstetric anesthesia (4). Many theories exist regarding the pathophysiology of PDPH, but it appears to be related to the loss of cerebrospinal fluid into the epidural space with a decrease in cerebrospinal fluid pressure and downward movement of the brain and traction on the dura (5). Spontaneous recovery within 5 days occurs in most cases, but PDPH can last up to many months, like the case report of Barbosa et al. demonstrated (6). During this time, patients suffer and rehabilitation is restricted. Pharmacological therapy is seldom a complete success (5), but an epidural blood patch can resolve the issue in many cases (7).

Due to the frequency of occurrence and the resulting physical limitations of patients, every effort should be made to learn about the risk factors of PDPH and how to avoid it. Nonmodifiable risk factors include gender, age, pregnancy, previous history of PDPH or chronic headache, and low body mass index (BMI). However, of modifiable risk factors, such as needle shape, bevel orientation, number of LP attempts, and prelumbar puncture positioning (8, 9), the most relevant appears to be needle size the smaller the size, the lower the PDPH incidence.

Nevertheless, the approach may be considerable. Median, paramedian, and Taylor´s approaches have been advocated as the primary method in different settings, whereas the median approach may be easier for trainees, because the primary orientation seems straightforward. If cerebrospinal fluid cannot be detected, many choose the paramedian approach as a second strategy. With regard to the angled paramedian approach as the primary method, Mossafa et al. show that there is no difference in the incidence of PDPH compared with the median approach. In their study, the number of attempts to puncture is restricted to only one. This may not reflect clinical reality but may be part of the reason for these interesting results, demonstrating this kind of approach is not a risk factor for PDPH.
